# Mr. Francis John Marshall

**Published:** 1911-04-08

**Authors:** 


Mr. Francis John Marshall, M.R.C.S., who had been for thirty years before his retirement from practice Resident Medical Officer at St. George's Hospital, has died at the age of sixty-eight. He was a student at St. Mary's and qualified in 1864. At St. Mary's he held several appointments, including those of registrar, house surgeon, and resident obstetric officer. He had also been R.M.O. at the Female Lock Hospital and Asylum.

